# Supplementary material for: Orientation-Locked DNA Origami for Stable Trapping of Small Proteins in the Nanopore Electro-Osmotic Trap
Source: Nano Lett. 2022 Dec 12;23(3):788–94. doi: 10.1021/acs.nanolett.2c03569 (PMC9912335; doi:10.1021/acs.nanolett.2c03569)
Supplement: Supplementary file 1 — nl2c03569_si_001.pdf [file nl2c03569_si_001.pdf]

Supporting Information

Orientation-locked DNA origami for stable trapping of small proteins in the NEOtrap

Chenyu Wen[1,2], Eva Bertosin[2], Xin Shi[2], Cees Dekker[2*], Sonja Schmid[1*]

1 NanoDynamicsLab, Laboratory of Biophysics, Wageningen University, Stippeneng 4, Wageningen, 6708 WE, The Netherlands.

2 Department of Bionanoscience, Kavli Institute of Nanoscience, Delft University of Technology, Van der Maasweg 9, Delft, 2629 HZ, The Netherlands.

*Corresponding author(s). E-mail(s): c.dekker@tudelft.nl, schmid@nanodynlab.org

Table of Contents

Note 1. Methods

Figure S1. Cholesterol functionalization positions in each design

Note 2. Docking of the bare or cholesterol-functionalized origami sphere onto pores

Figure S2. Current traces of docking of cholesterol-functionalized DNA origami spheres under a negative voltage ramp

Figure S3. Current traces of the undocking of bare DNA-origami spheres

Figure S4 Current traces of the open-pore with lipid bilayer coating

Figure S5. Current trace showing docking and undocking of a cholesterol-functionalized DNA-origami sphere

Figure S6. Flow rate distribution in the vertical and horizontal configurations of origami spheres

Figure S7. Current traces showing the controlled docking of DNA-origami spheres in a vertical or horizontal orientation

Figure S8. Trapping data of ovalbumin.

Note 3: Estimation of the viscous force on a trapped protein

Note 4. The trapping energy well

Figure S9. Trapping time of avidin proteins at different voltages by bare and cholesterol-functionalized DNA-origami spheres

Figure S10. Trapping Ribonuclease A by using a cholesterol-functionalized DNA-origami sphere or bare DNA-origami sphere

Figure S11. Current traces showing the trapping of different proteins by a bare origami sphere and a vertically locked cholesterol-functionalized origami sphere

Supporting note 1. Methods

Nanopore fabrication and ionic current measurement

The nanopores were drilled by a transmission electron microscope (Titan aberration-corrected TEM, Thermo Fisher Scientific, USA) in freestanding 20-nm-thick SiN_x membranes deposited on glass chips as described previously.[1] Glass chips were purchased from Goeppert LLC. (USA). Nanopore chips were rinsed with deionized water (DIW, Milli-Q®, Merck KGaA, Germany), acetone, ethanol, isopropanol, and DIW, in sequence as mentioned. Afterwards, they were further cleaned by oxygen plasma (SPI Supplies® Plasma Prep III™, USA) and then mounted in a custom-made polyether ether ketone (PEEK) flow cell with an electrolyte reservoir at each side of the nanopore and corresponding fluidic channels. The entire setup was placed in a Faraday cage to prevent electromagnetic interference during the electrical measurements. The electrolyte in the two reservoirs was electrically connected to an Axopatch 200B amplifier (Molecular Devices LLC, UK) by Ag/AgCl electrodes (silver wire chloridized in household bleach). Analog signals were digitalized by Digidata 1550B digitizer (Molecular Devices LLC, UK) and recorded on a computer with Clampex 10.5 software (Molecular Devices LLC, UK). After flushing both chambers with DIW, the chambers were filled with 1 M KCl for current-voltage (I-V) measurement (voltages ranging from -120mV to 120mV). The diameter of nanopores was extracted from their conductance by using the simple model as described in Ref. [2,3]. Unless stated differently, all measurements were performed under 500 kHz sampling, 100 kHz low-pass filter (four-pole internal Bessel filter), at room temperature of 21°C.

Lipid bilayer coating

In order to prevent the non-specific adsorption, surface passivation of the pore was implemented by using 1-palmitoyl-2-oleoyl-sn-glycero-3-phosphocholine (POPC, Avanti Polar lipids Inc., USA). Vials with POPC in chloroform were dried in a vacuum and subsequently stored at -20°C. Before usage, the lipids were re-suspended in 600KHM buffer (600mM KCl, 50mM HEPES, 5mM $MgCl_2$, pH 7.5) to a concentration of 1mg/ml. The suspensions were then sonicated in a bath sonicator (Branson, model: 1510, USA) for >20min. Then, 50µL of lipid suspension was added to the ground-side reservoir of the nanopore, while applying an AC voltage with triangle waveform, 50 mV peak amplitude, and 1 Hz frequency. Lipid bilayer coating of the pore decreases the pore conductance. After stabilization for 10min, the entire flow cell was totally immersed in DIW and the chambers were flushed with DIW with further incubation for 20 min. Then, the chambers were flushed with DIW again before the flow cell was taken out of the bath and dried externally, filled with 600KHM buffer, and reconnected to the amplifier. Finally, the I-V curve was measured again,

and compared with the I-V before coating, to extract the size of the nanopore before and after coating.

DNA-origami spheres and protein samples

The DNA-origami sphere was designed based on ref.[2] using cadnano2. The DNA-origami sphere was produced as previously described.[4] Oligonucleotides and scaffold were purchased through tilibit nanosystems GmbH (Munich). Briefly, the folding mixture contained a 7560-nucleotide long single-stranded DNA scaffold at a final concentration of 50 nM, staple strands at a final concentration of 175 nM (3.5× fold excess), and folding buffer (5 mM TRIS, 1 mM EDTA, 5 mM NaCl and 20 mM $MgCl_2$). The reaction mixtures were annealed in a T100 Thermal Cycler (Biorad) device using the following ramp: 65°C for 15 min, 60°C-40°C (1°C/hour). Afterwards, the reaction mixtures were incubated at room temperature. The reaction mixtures were purified by ultrafiltration, using Amicon Ultra 0.5 mL Ultracel filters (100k) and buffer containing 5 mM TRIS, 1 mM EDTA, 5 mM NaCl and 5 mM $MgCl_2$. First, the filters with 500 µL of buffer were centrifuged for 5 min at room temperature (RT) at 10,000 g. After removing the filtrate, 50 µL of the sample was diluted with 450 µL of buffer and centrifuged again for 5 min at RT at 10,000 g. 450 µL of buffer was added to the filters, which were centrifuged for 5 min at RT at 10,000 g. This step was repeated 3 times. For retrieving the sample, the filter was removed, placed upside-down in a new tube, and centrifuged for 5 min at RT at 10,000 g. The cholesterol-modified oligos were added at 4× excess per handle (total: 24× excess) overnight. Afterwards, the sample was purified by ultrafiltration using the same protocol as described above. Models of the structure were computed with CanDo [5] and rendered with ChimeraX.[6] In this study, docking was initiated by inserting 50µl of 20nM of DNA-origami sphere solution in 600KHM buffer into the cis chamber at positive voltage.

Avidin (from egg white) was purchased from Thermo Fisher Scientific (USA). ClpP was expressed and purified in-house as previously described.[7] dCas9 was purchased from New England Biolabs Inc. (USA). Other proteins were purchased as Gel Filtration Calibration Kits from Cytiva LLC. (USA). All proteins were dispersed in 600KHM buffer at the indicated concentrations.

Data processing

Data were analyzed using home-made code in MATLAB platform. For the detection of trapping events, the function '*findchangepts*' was adopted to find the time points of the current level changes between the docking state and trapping state. Information about each trapping event, including the duration, blockage amplitude, and interval between the two adjacent events, could be extracted. In

addition, an amplitude threshold for trapping event detection was placed at three times the standard deviation of the noise of the docking baseline. In order to estimate the uncertainties of kinetic rate constants (i.e. trapping time), bootstrap sampling was used: for a set of n data points, 10 subsets with a size of 60% of n were randomly picked with replacement, fitted by an exponential distribution separately. Then, the means and standard deviations of the extracted trapping time across these subsets are calculated.

COMSOL simulations

The numerical simulations of the NEOtrap system were implemented on COMSOL Multiphysics 5.4 with a two-dimensional axial symmetrical domain. The simulations included the fluid, the membrane, and the DNA-origami sphere, whose relative permittivity was set to 80, 7.5,[8] and 8.3,[9] as deduced for water, SiN_x, and DNA, respectively. The ion distribution and movement in an electrolyte were governed by the Nernst−Planck equation, the electric potential distribution was described by the Poisson equation, and the fluid flow was determined by the Navier−Stokes equations. The *Transport of Diluted Species* module (Nernst−Planck equation), the *Electrostatics* module (Poisson equation), and the *Laminar Flow* module (Navier−Stokes equations) were incorporated and fully coupled in the simulation. The electrolyte was 600 mM KCl with the mobilities of K^+ and Cl^- were 7.0×10^{-8} and 7.2×10^{-8} $m^2\ V^{-1}\ s^{-1}$, respectively.[10] The respective diffusion coefficient was then determined through the Einstein relation. It is worth noting that in order to reduce the demand for computation resources and to reach a converged model, a two-dimensional axial symmetry was adopted instead of a full three-dimensional simulation.

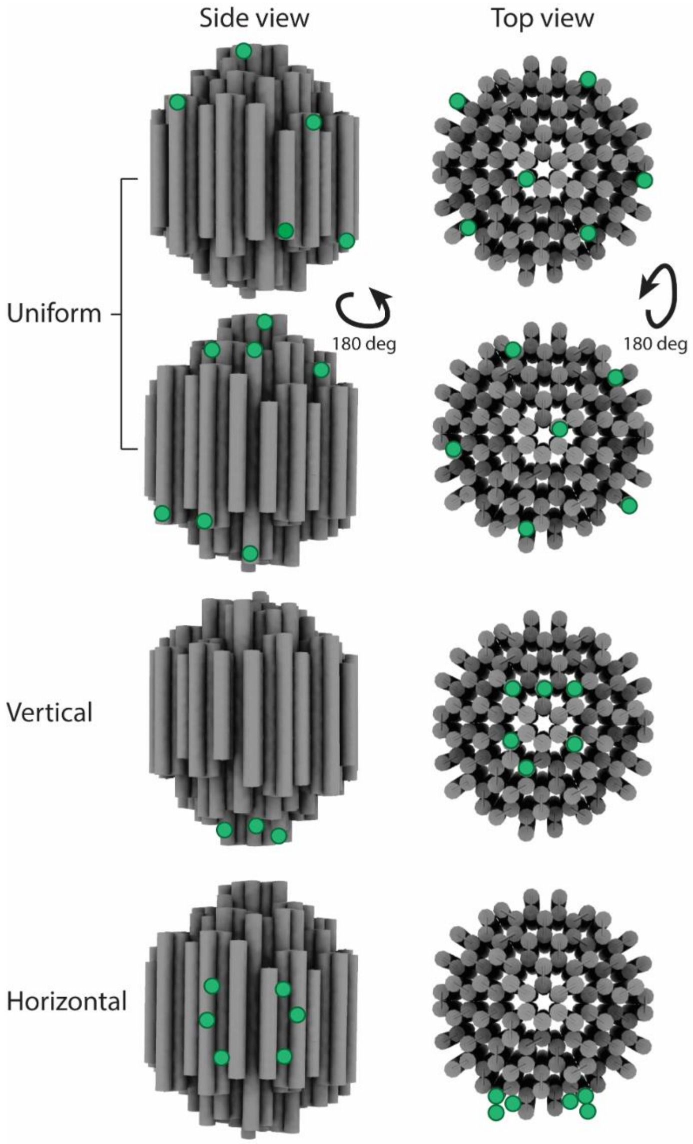

Figure S1. Cholesterol functionalization positions in each origami design. The green dots represent the attachment points of the cholesterol molecule.

Supporting Note 2: Docking of the bare or cholesterol-functionalized origami sphere onto the pores.

In order to measure the strength of the interaction between the cholesterol anchors and the lipid bilayer, we linearly decreased the voltage and recorded the corresponding current traces. As shown in Fig. S2(a), docking of a single origami sphere happened in a holding period with a constant 100 mV bias. After docking, the voltage was ramped down from 100 mV to -500 mV. Beyond a certain negative voltage, the noise suddenly increased, indicating that the lipid bilayer became unstable, and the sphere undocked under the high electric field. No distinct changes were observed until this voltage (in contrast to the data for bare pores, cf. Fig. S4). The voltages causing the instability of the lipid bilayer were regarded as the release voltage of the cholesterol-functionalized origami spheres.

Compared to the cholesterol-functionalized origami sphere, the bare origami spheres displayed different current traces during a negative voltage ramp. As shown in Fig. S3, a sudden current increase was observed at about positive +50 mV, which was caused by the undocking of the sphere whereupon the current returned to the open pore level. Thus, for a bare origami sphere, even a small positive voltage was insufficient to stably hold the sphere on the pore, which we attribute to thermal fluctuations.

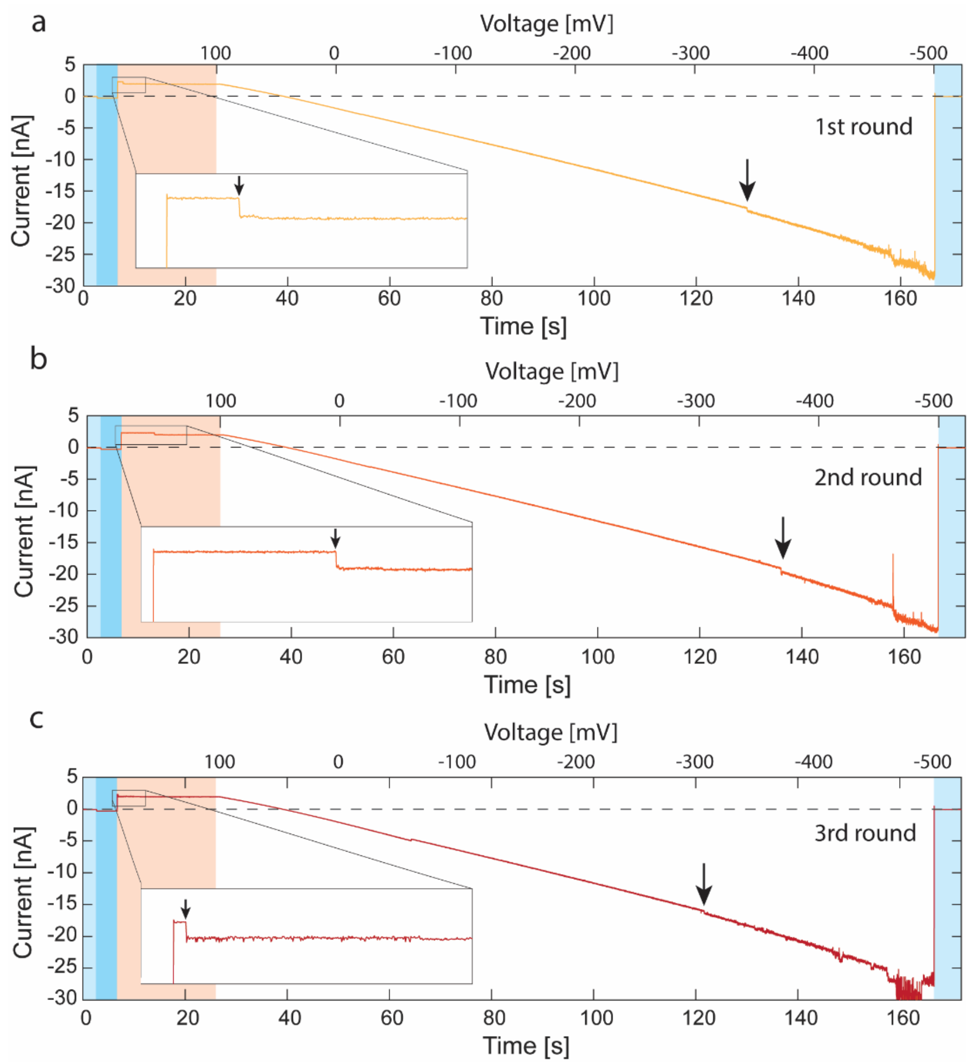

Figure S2. Current traces of the docking and undocking of cholesterol-functionalized DNA-origami spheres under a negative voltage ramp. (a-c) Traces of the 1st, 2nd, and 3rd rounds of the voltage cycle. In the light blue region, the bias voltage was zero. Then, -20 mV was applied to repel any possible docked sphere, marked as the dark blue color. Afterwards, +100 mV bias was added for 15s to attract origami spheres and dock one sphere onto the nanopore, for a period marked in the orange color. In this period, a current drop was observed, which is caused by the docking of an origami sphere due to the steric blockage of the pore, as indicated by the arrow in the zoomed-in view of the inset. Next, the bias voltage was ramped down linearly from 100 mV to -500 mV. A gradual change of current was observed correspondingly. At a certain negative voltage as marked by the arrow, the current noise suddenly increased. We attribute this to a sudden instability of the lipid bilayer induced by the high electric field, and the point where the cholesterol anchors of the origami sphere are likely pulled out from the lipid bilayer to release the sphere which is electrophoretically pulled away from the pore. In the next round, the lipid bilayer resealed and a new docking by another origami sphere could be studied. A similar pattern appeared every round of the repeated cycles, as shown in (a) to (c).

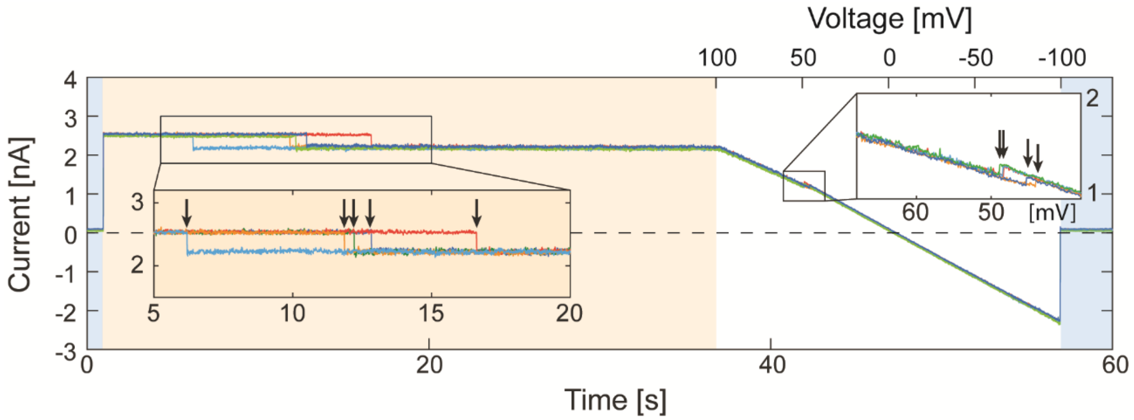

Figure S3. Current traces of the undocking of bare DNA-origami sphere. Voltage cycles were repeated five times and the corresponding current traces are shown with different colors. In the light blue region, the bias voltage was zero. Then, +100 mV bias was added for 36s (orange color) to attract an origami sphere and dock it on the nanopore, leading to the observed current drop, as indicated by the arrows in the zoomed-in view of the left inset. Afterwards, the bias voltage was ramped down gradually from 100 mV to -100 mV, and a gradual decrease in the current was observed. At a certain voltage of about +50 mV (marked by the arrows in the zoomed-in view of the right inset, the horizontal axis is voltage) the current suddenly increased, indicating the undocking of the sphere. A similar pattern was found in every round of the voltage cycles.

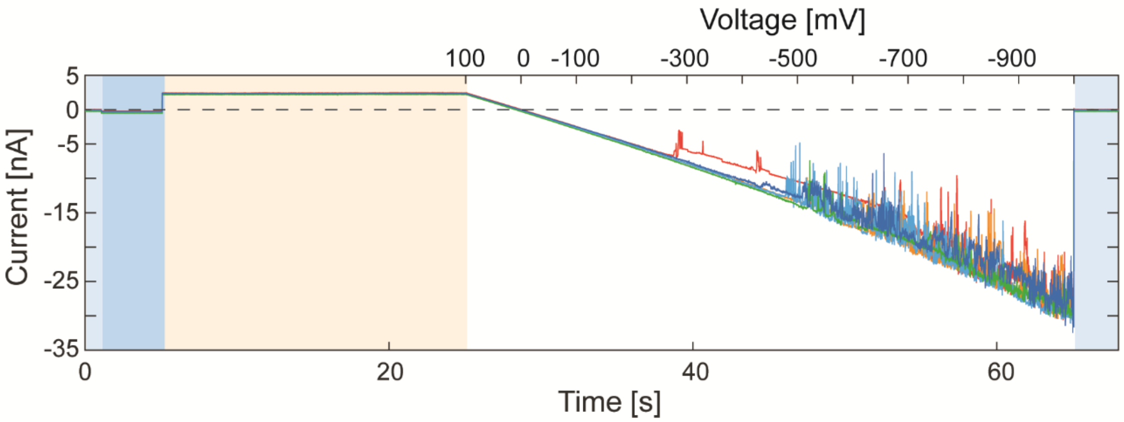

Figure S4. Current traces of the open-pore with lipid bilayer coating under a negative voltage ramp. Voltage cycles were repeated five times and the corresponding current traces are shown in different colors. The voltage cycle was similar to that in Figure S1. The only quantitative difference was that the voltage decreased from 100 mV to -1000 mV. Similar to the traces in Figure S1, the noise suddenly increased at a certain negative voltage, indicating an unstable lipid bilayer. This suggests that the origami sphere locked by the cholesterol molecules is only able to undock if the lipid bilayer becomes unstable.

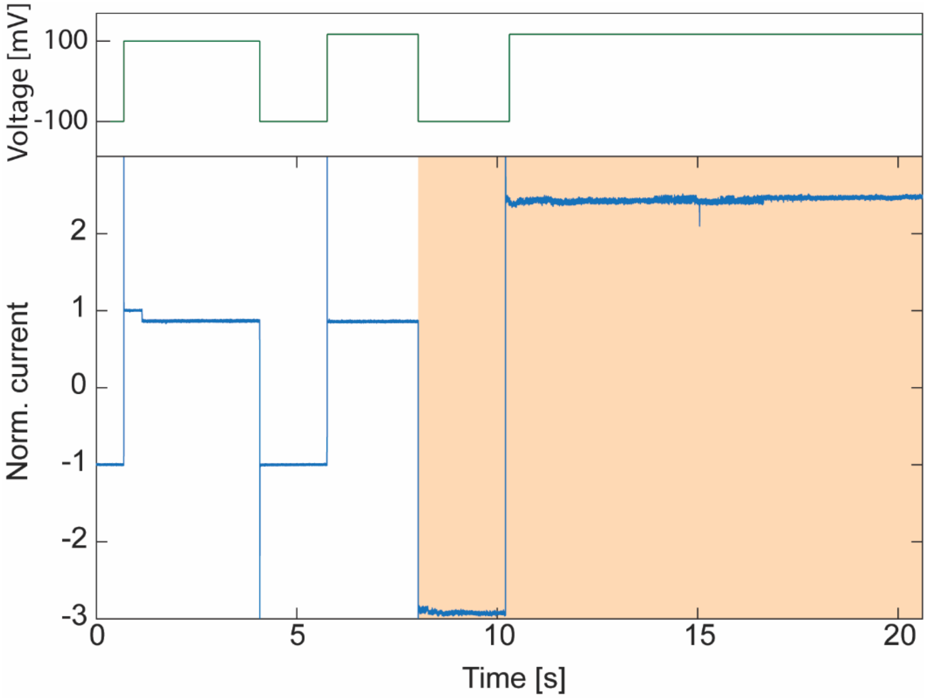

Figure S5. Current trace showing docking and undocking of a cholesterol-functionalized DNA-origami sphere. The bottom panel shows the current versus time. The corresponding profile of the applied voltage is shown in the upper panel. When a 100 mV bias was applied, a current decrease step was found, indicating the docking of an origami sphere. Since the cholesterols anchored the sphere onto the nanopore, the current was kept at the docking level upon switching the voltage to ±100 mV. Finally (orange region), application of -100 mV bias removed the sphere and peeled off the lipid bilayer, yielding a very high current that indicates a bare pore without a lipid bilayer.

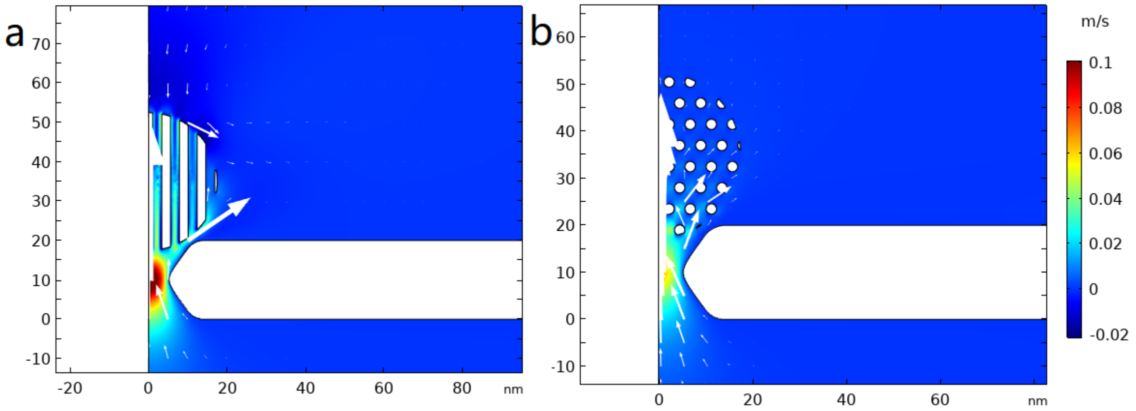

Figure S6. Flow rate distribution in the (a) vertical and (b) horizontal configuration of the origami sphere. The arrow angles indicate the direction of the flow and their length is proportional to the magnitude of flow rate. Color represents the vertical component of the flow velocity with the positive direction pointing upwards.

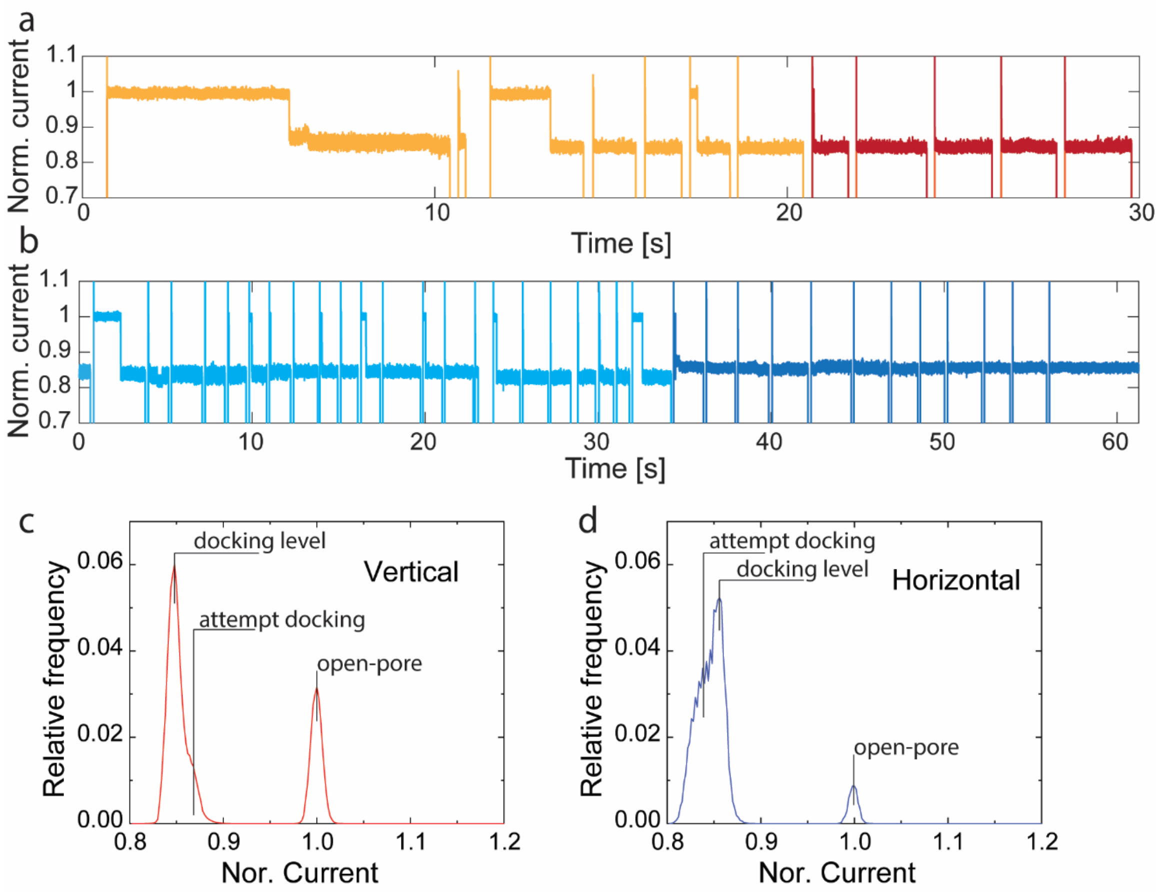

Figure S7. Current traces showing the controlled docking of DNA-origami spheres in a vertical or horizontal orientation. (a) Current trace of vertical sphere docking. Here, cholesterol molecules were only functionalized at the bottom of the origami sphere. As shown in yellow color, a current drop was observed after every voltage inversion in a series of alternating voltages, indicating the sphere was not well locked by the cholesterol. However, in the voltage cycle at 21 s (red color), the sphere was docked 'irreversibly' (i.e. presumably stably locked by cholesterol), in such a way that the negative voltage did not remove the sphere. Instead, the current stayed at the docking level after following voltage inversions. (b) Similar current traces for horizontal sphere docking. The sphere was locked by the cholesterols in the voltage cycle at 34 s whereupon afterwards, the current stayed at the docking level shown in the dark blue color. (c) Current distribution of the vertical docking, as deduced from the trace in panel a. (d) Same as c but for horizontal, based on trace in panel b. For the vertical configuration, the current level of stable docking locked by cholesterols was lower than the level of attempt temporary docking. The reversed phenomenon was observed for the horizontal configuration, i.e., the locked docking level was higher than the attempt docking level. We attribute this to the non-isotropic sphere geometry which allows the vertically oriented sphere to reach deeper into the pore – and thus block more through-pore current – than the horizontally oriented sphere.

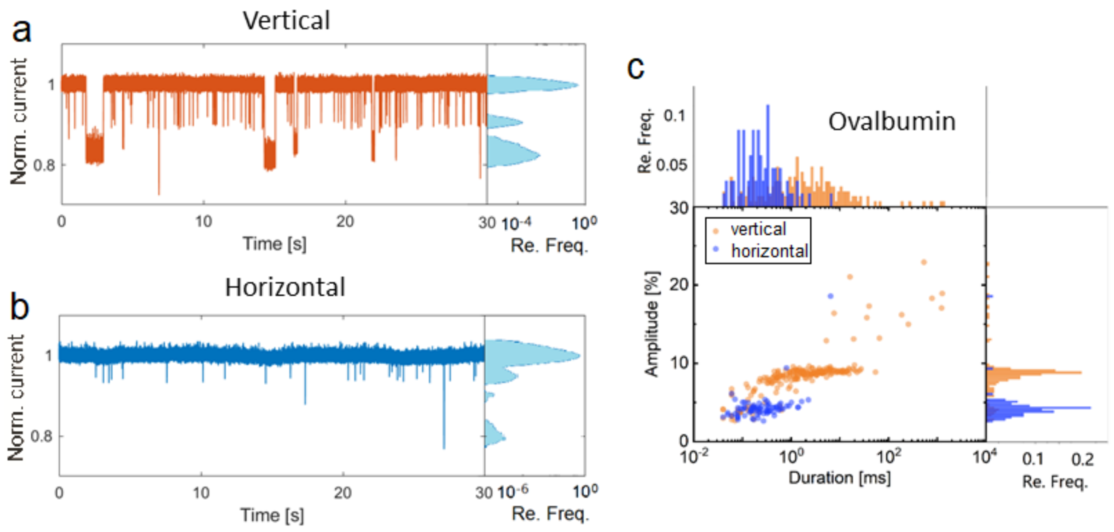

Figure S8. Trapping date of ovalbumin. Current traces show the trapping of ovalbumin by vertically (a) and horizontally (b) docked origami spheres at 100 mV bias. The histograms of the corresponding current traces are shown on the right. (c) Scatter plots compare the trapping time and relative blockage amplitude of the trapping events from panels a and b.

Supporting Note 3: Estimation of the viscous force on a trapped protein

According to Stokes' law, a viscous sheer force acting on a sphere can be calculated by

$$F_v = 6\pi\eta v_{eof} r$$

where η is the viscosity of water, v_{oef} is the water velocity, and r is the radius of the sphere. Approximating the shape of proteins by a sphere, the viscous force to hold a trapped protein in the pore can thus be estimated.

From the simulation, the maximum velocity of the EOF in the pore is around 0.1 m/s for the vertically locked origami sphere (Figure 3b in the main text). The radius of an avidin molecule is ~3 nm. Thus, the approximate viscous force to hold the trapped avidin is in the single-digit pico-newton range:

$$F_v = 6\pi \times 10^{-3}[Pa \cdot s] \times 0.1[m/s] \times 3 \times 10^{-9}[m] = 5.6[pN]$$

Supporting Note 4: The trapping energy well

The escape from the trapping potential well can be described as an energy barrier-crossing process. The escape rate k_{off} (*i.e.*, the reciprocal of the trapping time τ_{trap}) then follows an Arrhenius relationship with the energy barrier height,

$$k_{off} = \frac{1}{\tau_{trap}} = k_0 \exp(-\frac{E_b}{k_B T})$$

where, k_0 is a rate constant, E_{b} is the trapping energy barrier height, k_B is the Boltzmann constant, and T is the absolute temperature. Thus, a prolonging of the trapping time indicates the increase of the energy barrier of escape,

$$A = \frac{\tau_{trap,2}}{\tau_{trap,1}} = \exp(\frac{E_{b,2} - E_{b,1}}{k_B T}) = \exp(\frac{\Delta E}{k_B T})$$

where A is the ratio of prolonged trapping, and ΔE the corresponding increase of the barrier height. An increase of the trapping time by a factor 100 thus means an increase of 4.6 $k_{\mathrm{B}}T$ in barrier height.

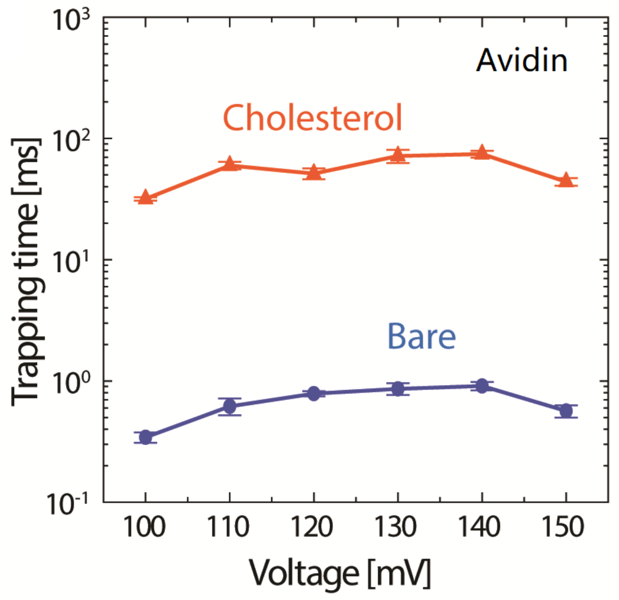

Figure S9. Trapping time of avidin proteins at different voltages by bare and cholesterol-functionalized DNA-origami spheres. The error bars show the standard deviation of extracted parameters from fitting by using bootstrap sampling.

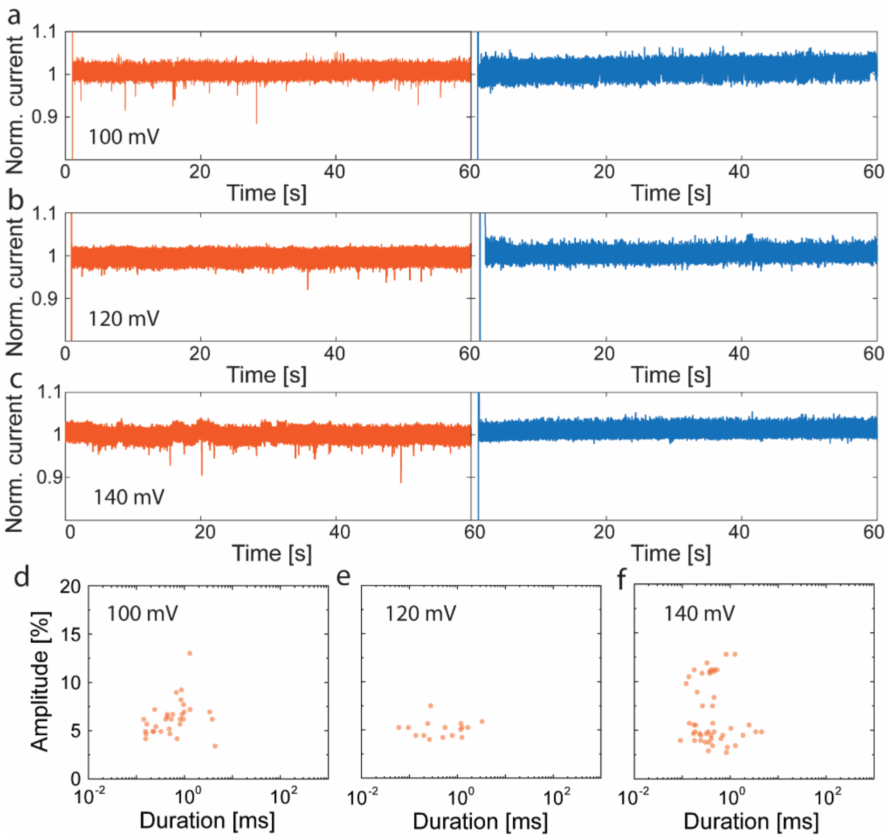

Figure S10. Trapping of Ribonuclease A (13.7 kDa) using a cholesterol-functionalized or bare DNA-origami sphere. (a-c) Typical current traces of trapping by a cholesterol-functionalized origami sphere (left panel) or a bare origami sphere (right panel) at 100 mV, 120 mV, and 140 mV, respectively. (d-f) Scatter plots showing the trapping time and relative blockage amplitude of the trapping events of Ribonuclease A for cholesterol-functionalized origami spheres at different voltages from (a) to (c). No trapping events were observed with the bare origami sphere.

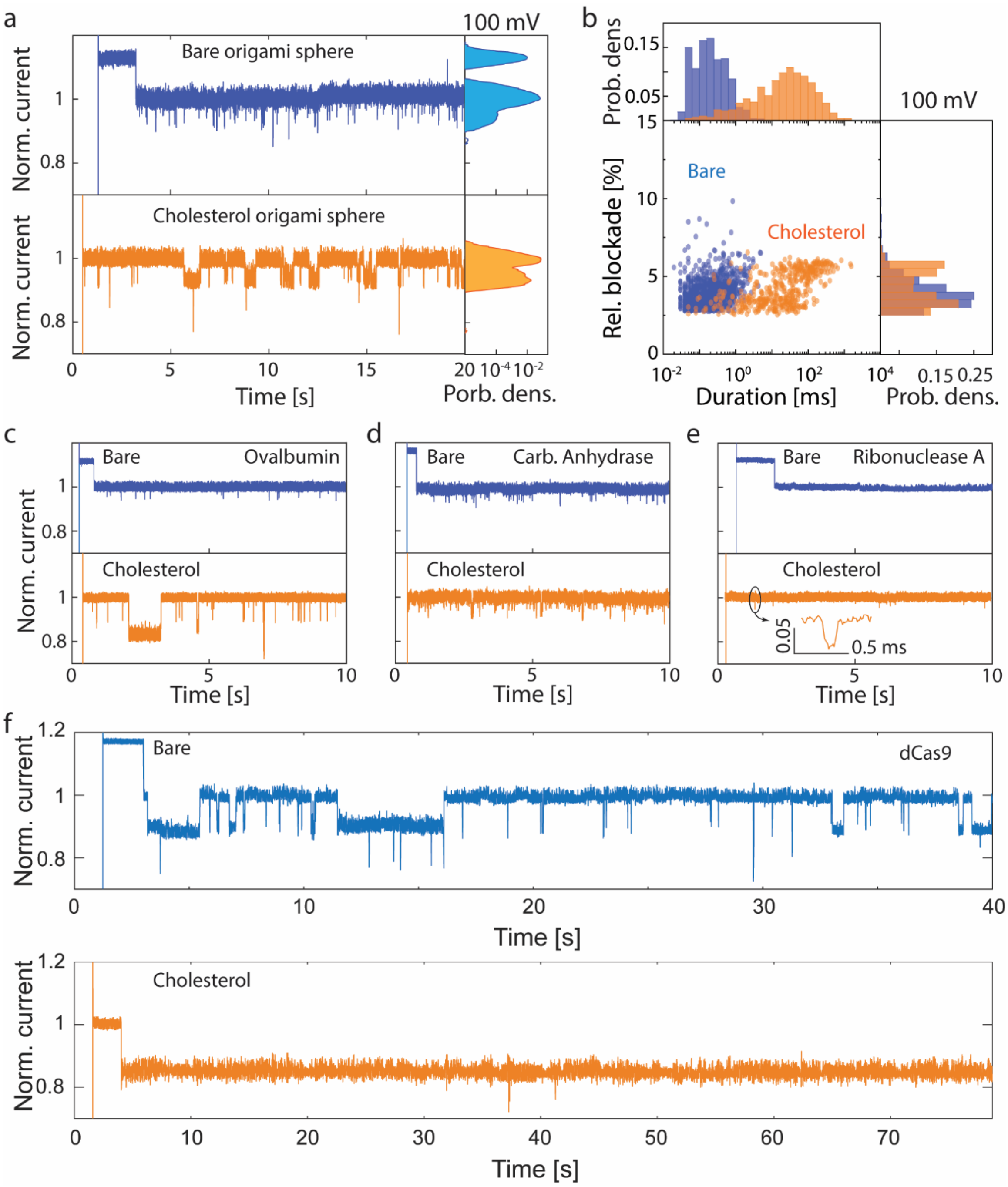

Figure S11. Current traces showing the trapping of different proteins by a bare origami sphere and a vertically locked cholesterol-functionalized origami sphere at 100 mV bias. (a) Trapping of avidin at 100 mV. Histograms of the corresponding current traces are shown at the right-hand side. (b) Scatter plot that compares the trapping time and relative blockage amplitude from data of panel a. (c-f) Current traces of trapping ovalbumin (c), carbonic anhydrase (d), ribonuclease A (e), and dCas9 (f) for both bare origami spheres (blue) and cholesterol-functionalized origami spheres (orange) at 100 mV.

References:

(1) van den Hout, M.; Hall, A. R.; Wu, M. Y.; Zandbergen, H. W.; Dekker, C.; Dekker, N. H. Controlling Nanopore Size, Shape and Stability. *Nanotechnology* **2010**, *21* (11), 115304. https://doi.org/10.1088/0957-4484/21/11/115304.

(2) Schmid, S.; Stömmer, P.; Dietz, H.; Dekker, C. Nanopore Electro-Osmotic Trap for the Label-Free Study of Single Proteins and Their Conformations. *Nat. Nanotechnol.* **2021**, *16* (11), 1244–1250. https://doi.org/10.1038/s41565-021-00958-5.

(3) Kowalczyk, S. W.; Grosberg, A. Y.; Rabin, Y.; Dekker, C. Modeling the Conductance and DNA Blockade of Solid-State Nanopores. *Nanotechnology* **2011**, *22* (31), 315101. https://doi.org/10.1088/0957-4484/22/31/315101.

(4) Wagenbauer, K. F.; Engelhardt, F. A. S.; Stahl, E.; Hechtl, V. K.; Stömmer, P.; Seebacher, F.; Meregalli, L.; Ketterer, P.; Gerling, T.; Dietz, H. How We Make DNA Origami. *ChemBioChem* **2017**, *18* (19), 1873–1885. https://doi.org/10.1002/cbic.201700377.

(5) Kim, D.-N.; Kilchherr, F.; Dietz, H.; Bathe, M. Quantitative Prediction of 3D Solution Shape and Flexibility of Nucleic Acid Nanostructures. *Nucleic Acids Res.* **2012**, *40* (7), 2862–2868. https://doi.org/10.1093/nar/gkr1173.

(6) Pettersen, E. F.; Goddard, T. D.; Huang, C. C.; Meng, E. C.; Couch, G. S.; Croll, T. I.; Morris, J. H.; Ferrin, T. E. UCSF CHIMERAX : Structure Visualization for Researchers, Educators, and Developers. *Protein Sci.* **2021**, *30* (1), 70–82. https://doi.org/10.1002/pro.3943.

(7) van Ginkel, J.; Filius, M.; Szczepaniak, M.; Tulinski, P.; Meyer, A. S.; Joo, C. Single-Molecule Peptide Fingerprinting. *Proc. Natl. Acad. Sci.* **2018**, *115* (13), 3338–3343. https://doi.org/10.1073/pnas.1707207115.

(8) Sze, S. . M.; Ng, K. K. Appendix H:Properties of SiO2 and Si3N4. In *Physics of Semiconductor Devices*; A JOHN WILEY & SONS, JNC.: New Jersey, 2007; p 791.

(9) Cuervo, A.; Dans, P. D.; Carrascosa, J. L.; Orozco, M.; Gomila, G.; Fumagalli, L. Direct Measurement of the Dielectric Polarization Properties of DNA. *Proc. Natl. Acad. Sci.* **2014**, *111* (35). https://doi.org/10.1073/pnas.1405702111.

(10) A. W., A. Chapter 13: Electrochemical Cells. In *A Textbook of Physical Chemistry*; Academic Press: New York, 1973; p 506.
